# Post-discharge care following acute kidney injury: quality improvement in primary care

**DOI:** 10.1136/bmjoq-2019-000891

**Published:** 2020-12-16

**Authors:** Susan J Howard, Rebecca Elvey, Julius Ohrnberger, Alex J Turner, Laura Anselmi, Anne-Marie Martindale, Tom Blakeman

**Affiliations:** 1 NIHR Applied Research Collaboration Greater Manchester (ARC-GM), Health Innovation Manchester, Manchester, UK; 2 Centre for Primary Care and Health Services Research, Division of Population Health, Health Services Research and Primary Care; School of Health Sciences, Faculty of Biology, Medicine and Health, The University of Manchester, Manchester, UK, The University of Manchester, Manchester, UK; 3 Department of Infectious Disease Epidemiology, Imperial College London, London, UK; 4 Health Organisation, Policy and Economics (HOPE) group, Centre for Primary Care and Health Services Research, The University of Manchester, Manchester, UK

**Keywords:** acute kidney injury, patient safety, primary care, audit and feedback, clinical audit

## Abstract

**Background:**

Over the past decade, targeting acute kidney injury (AKI) has become a priority to improve patient safety and health outcomes. Illness complicated by AKI is common and is associated with adverse outcomes including high rates of unplanned hospital readmission. Through national patient safety directives, NHS England has mandated the implementation of an AKI clinical decision support system in hospitals. In order to improve care following AKI, hospitals have also been incentivised to improve discharge summaries and general practices are recommended to establish registers of people who have had an episode of illness complicated by AKI. However, to date, there is limited evidence surrounding the development and impact of interventions following AKI.

**Design:**

We conducted a quality improvement project in primary care aiming to improve the management of patients following an episode of hospital care complicated by AKI. All 31 general practices within a single NHS Clinical Commissioning Group were incentivised by a locally commissioned service to engage in audit and feedback, education training and to develop an action plan at each practice to improve management of AKI.

**Results:**

AKI coding in general practice increased from 28% of cases in 2015/2016 to 50% in 2017/2018. Coding of AKI was associated with significant improvements in downstream patient management in terms of conducting a medication review within 1 month of hospital discharge, monitoring kidney function within 3 months and providing written information about AKI to patients. However, there was no effect on unplanned hospitalisation and mortality.

**Conclusion:**

The findings suggest that the quality improvement intervention successfully engaged a primary care workforce in AKI-related care, but that a higher intensity intervention is likely to be required to improve health outcomes. Development of a real-time audit tool is necessary to better understand and minimise the impact of the high mortality rate following AKI.

## Problem

Acute kidney injury (AKI) has become a major barometer for assessing severity of acute illness and to drive improvements in patient safety and health outcomes.^1–8^ Building on the implementation of national policy in England for hospital care, a quality improvement project was conducted to improve the primary care identification and management of patients following an episode of hospital care complicated by AKI.

The quality improvement project was delivered by The National Institute for Health Research Collaboration for Leadership in Applied Health Research and Care Greater Manchester (NIHR CLAHRC GM; a regionally based partnership between providers of NHS services and universities) in collaboration with NHS Bury Clinical Commissioning Group (CCG; an NHS body responsible for the planning and commissioning of healthcare services for its local area). NHS Bury CCG covers an urban area in Greater Manchester, with a population of approximately 190 000 patients. The AKI project was carried out in the context of NHS Bury CCG developing a locally commissioned service to implement regional primary care standards to reduce variation in care and improve health outcomes.^9 10^

The improvement project directly built on an incentivised hospital AKI improvement activity conducted within the local hospital, which sought to improve discharge summaries for patients following AKI.^11^ However, there was a lack of data to understand post-discharge management in primary care. Aligned with national priorities, over a 3-year audit period, the project focused on measuring and improving four key clinical recommendations in general practice: diagnostic AKI coding; conducting a medicines review; monitoring kidney function; and provision of written information to patients following AKI.^1 11–14^

## Background

AKI is a clinical syndrome that is characterised by a sudden reduction in kidney function associated with episodes of acute illness.^2^ Patients who experience illness complicated by AKI are at a significantly higher risk of worse health outcomes in the short to long term, including higher risk of AKI recurrence, development or progression of chronic kidney disease up to end-stage renal disease, and premature mortality.^2 15 16^ There is evidence that AKI is a ‘strong, consistent and independent risk factor’ for unplanned hospital readmission.^17 18^ Hospital-related care for patients with AKI is estimated to cost around 1% of the NHS budget.^3^

Over the past decade, AKI quality improvement initiatives have largely focused on management in hospital.^1 19–21^ In England, this was influenced by the National Confidential Enquiry into Patient Death and Outcome 2009 Report on AKI, which identified significant hospital failings in patient safety in terms of poor assessment of acute illness and delays in recognising AKI, with evidence to suggest that approximately one in five episodes of AKI were avoidable.^22^ However, recognising evidence that AKI is of relevance across all health and care settings, there is a shift to broaden AKI quality improvement efforts across the interface and into primary care.^7 15 23–26^

In 2015–2016, hospitals in England were financially incentivised to improve discharge care following AKI.^11^ The stated goal was “to improve the follow up and recovery for individuals who have sustained AKI, reducing the risks of readmission, re-establishing medication for other long term conditions and improving follow up of episodes of AKI, which is associated with increased cardiovascular risk in the long term”.^11^ Addressing gaps in discharge communication, it aimed to develop the knowledge base of primary care practitioners on AKI and to “positively impact on readmission rates for patients with AKI”.^11 27^ Payments were made to hospitals for documentation of four key items on a patient’s discharge summary: (1) stage of AKI; (2) evidence of a medicines review having been undertaken; (3) type of blood tests required on discharge; and (4) frequency of blood tests required on discharge for monitoring.^11^

Although identified as a priority, to date there is limited AKI quality improvement work in the primary care setting or evidence of its impact. Therefore, the project aligned with local (NHS Bury CCG),^10^ regional (Greater Manchester standards)^9^ and national (NHS England Think Kidneys; a national programme of NHS England to improve the care of people at risk of, or with, AKI) priorities.^7 11^ The project built on the introduction of hospital-based incentives and aimed to develop a quality improvement model to understand and address gaps in post-discharge AKI care in primary care. Process and impact evaluations were conducted in parallel to maximise learning.

## Measurement

A clinical audit was conducted to track changes in key indicators of processes of care. Data were collected manually by the project team for three consecutive financial years from April 2015 to March 2018. We audited all patients who (1) had an admission in the local hospital and who had been given a clinical diagnosis of AKI (chapter N17 of the International Classification of Diseases version 10 (ICD-10)),^28^ (2) had AKI noted on their hospital discharge summary and (3) were still active on primary care patient records systems (excluding those who had left the practice or were deceased) at the time of the audit.

The selected measures were aligned with national guidance and National Institute for Health and Clinical Excellence (NICE) pilot indicators.^13 29 30^ They focused on four key processes of care: (1) recording of AKI diagnosis by Read coding (a diagnosis and administrative coding system used in primary care) on general practice patient records (Read codes K04.12, K04C.00, K04E.00 and K04D.00); (2) medication review undertaken within 1 month (31 days) of discharge from hospital; (3) serum creatinine check (to measure kidney function) undertaken within 3 months (93 days) of discharge from hospital; and (4) written information about AKI given to patients (Read code 8OAG). For time-sensitive measures, we counted from the date of discharge as this is the first opportunity for primary care teams to be aware/act.

In addition to the clinical audit, an outcomes evaluation was also conducted for patients who were discharged following a hospital stay complicated by AKI. We assessed changes in healthcare outcomes, measured at the patient level and risk adjusted for demographics and comorbidities, and whether these differ across patients registered with primary care practices with better processes of care. We examined changes in hospital unplanned readmission within 30 and 90 days from discharge after an admission including an AKI complication, as a measure of improved management in primary care; mortality within 30 and 90 days after an AKI episode; and average length of stay at first readmission within 90 days as well as the total number of bed days across all readmissions within 90 days from discharge, as proxies of the severity and financial consequences of the readmission. We used Secondary Use Services (SUS; a single comprehensive repository for healthcare data in England)^31^ data from 1 April 2014 to 31 March 2016 which served as a 2-year ‘pre-intervention’ period, and April 2016 (when the locally commissioned service started)^10^ to March 2018 served as a combined ‘implementation plus post-intervention period’. We used a controlled before-and-after study design and a difference-in-difference identification strategy.^32 33^ We compared outcomes for patients from NHS Bury CCG, where the intervention had taken place (‘treatment group’), with outcomes for patients from three neighbouring CCGs treated at the same hospital, but not exposed to the primary care intervention (‘comparator group’). Trends in outcomes in the intervention and comparator group before the implementation of the intervention were parallel; therefore, changes in outcomes in the comparator group reflect changes in the treatment group had the intervention not been implemented. Consequentially, differences in outcomes in the treatment group can be attributed to the intervention.

A qualitative process evaluation was also conducted alongside this study to aid understanding of AKI-related working practices in primary care, the data from which are published separately.^34^ Elements of these findings have been included in this report where relevant.

## Design

This intervention was co-designed with the CCG and was informed by an evidence base to suggest interventions that combine professional education, audit and feedback, with financial incentives have the potential to change professional behaviour and improve patient safety in primary care.^35–39^ Through a locally commissioned service (NHS Bury CCG Quality in Primary Care Contract),^10^ general practices were incentivised to (1) participate in an audit of coding of AKI, (2) attend a multidisciplinary professional education training session, and (3) develop and implement a practice-level action plan to improve management of AKI in primary care.

### AKI educational events

Inclusion of an educational element to interventions has been shown to change clinical practice.^40^ This was a critical element to raise awareness of AKI, share best practice, and highlight the need and potential benefit of primary care input. Participants were provided with resources developed through NHS England’s Think Kidneys Programme, such as the information leaflet for patients and carers.^7^ We co-delivered the 2-hour events with NHS Bury CCG, which comprised presentations and interactive discussion and exercises focused on developing an AKI action plan. Participants from different practices worked together in groups to share learning. In addition to local primary care leadership to support engagement and help place AKI in clinical context, we purposefully involved clinical experts including a renal consultant based at a regional hospital.

### Audit and feedback

There is evidence that targeted audit and feedback interventions are deemed to more effective when there is a focus on areas of low baseline performance, it is delivered in both verbal and written formats, and when it includes explicit targets and an action plan.^36 37 39^ The records of active patients registered with a primary care practice in NHS Bury CCG who had been discharged from the local hospital following an episode of illness complicated by AKI were audited over a 3-year period (2015–2018). Audit data were collected, analysed and a practice-level report was fed back to each practice, comparing with anonymised CCG-wide data. This provided a measure of (1) how their practice had improved individually since the previous visit and (2) how they compared with their local peers. Within the reports, top areas for improvement were highlighted.

Initially, supported by data from an informatics tool, it was proposed that quarterly feedback visits would be carried out by the project team. However, due to challenges around the development of an informatics tool, this was altered to annual face-to-face visits, with audit data collected manually on an annual basis. Credibility of accurate data is essential to ensure buy-in, particularly in the context of an emerging clinical area.^41^ Collating data where AKI has not been coded (or when one of the many possible alternative codes has been used) on primary care practice systems is a key factor that needs to be considered for follow-on studies. In response to the first audit data, practices were asked to review and (where appropriate) clinically code the patients identified. Subsequent audit and feedback visits aimed to sustain and enhance improved coding and management of this patient population. During these visits, and through email/phone contact between times, relationships were built with primary care practices (usually via an identified point of contact who was engaged with the work) by the project team to support ongoing project delivery. This was reinforced by a new team of practice pharmacists who became enthusiastic about the project as it resonated with their clinical practice; they helped facilitate practice engagement and often delivery of the improvement work.

Primary care practices were supported to develop their own practice-level action plans through the educational events, provision of resources, an initial audit/feedback visit, as well as an offer of further support from the project team. Action plans aimed to improve the management of this patient population by focusing on the key processes of care.

### Financial incentives

Although financial incentives have been shown to have variable effects on processes of care, they have the potential to enable engagement in quality improvement activities.^42^ The way in which this was delivered (in the form of standards within a locally commissioned service) aimed to provide alignment with local priorities and demonstrated clear CCG support for the intervention (cited as a driver by practices), and therefore was expected to facilitate change. Following the education events, practice representatives were requested to disseminate the learning back to their primary care teams, and it was suggested they do this through an established monthly, CCG-financially supported, local learning time initiative.

### Patient and public involvement

We involved patients/public during this project to explore how they might input to the study, and they also reviewed the participant facing materials.

## Strategy

We chose to take a flexible and reactive quality improvement approach due to the recognised capacity limitations of the primary care practices involved, thus allowing for adjustments throughout the course of the project and individualisation per site. The intervention was designed in collaboration between the NIHR CLAHRC GM team and key stakeholders at NHS Bury CCG, and informed by evidence plus cumulative experiential learning from years of delivering quality improvement work in primary care. The longitudinal analysis limited alteration of the defined audit measures, but these were carefully thought through prior to commencement and were intentionally aligned with current national guidance.^29^ The detail and justification for choice of design and any subsequent alteration is detailed in the prior section; ultimately, the original design was delivered as planned, with the exception of the shift to manual audit and feedback, with practice visits conducted on an annual basis.

The AKI improvement work coincided with an increase in the practice pharmacist workforce in general practices in Bury as part of wider recruitment initiatives. The level of engagement and input varied per practice. Some pharmacists played a key role in planning and implementing the intervention, including writing action plans, acting as an information resource within practices, as well as identifying cases of AKI, coding an AKI diagnosis and undertaking medication reviews. Becoming more integrated into practice teams as part of this work, and having access to clinical information about patients, facilitated some pharmacists to extend the scope of their professional roles beyond the remit of this work (an unintended benefit of the work). However, others felt constraints of their roles limited potential involvement in care for patients following AKI.^34^

## Results

Five educational events were delivered at the start of the intervention period, with all 31 general practices represented. The total number of participants across the events was 82, of which 64 were practice staff (primary care practitioners (GPs), nurses, practice managers and administrators), 10 were pharmacists employed in practices and 8 were CCG medicines optimisation pharmacists (5 of whom attended more than one event). The analysis of our qualitative data showed that the events were generally well received.^34^ Participants reported that after attending, they felt better equipped to implement the intervention. GPs and practice managers welcomed the opportunity to find out how their peers in local practices were approaching implementation, while pharmacists particularly appreciated the clinical content which helped increase their knowledge about kidney health.^43^

### Processes of care

Through the audit period, around 1500 patients per year were clinically coded with AKI (ICD-10 N17) and discharged from the local hospital. Of these, approximately 60% were excluded from the analysis as the patients were no longer active on the system (left the practice or deceased), there was no discharge summary available on the practice system or there was no mention of AKI on the discharge summary. In total, over the 3 years, we audited 1669 patient records (431 from 2015 to 2016, 633 from 2016 to 2017, and 605 from 2017 to 2018).

**Figure 1:**
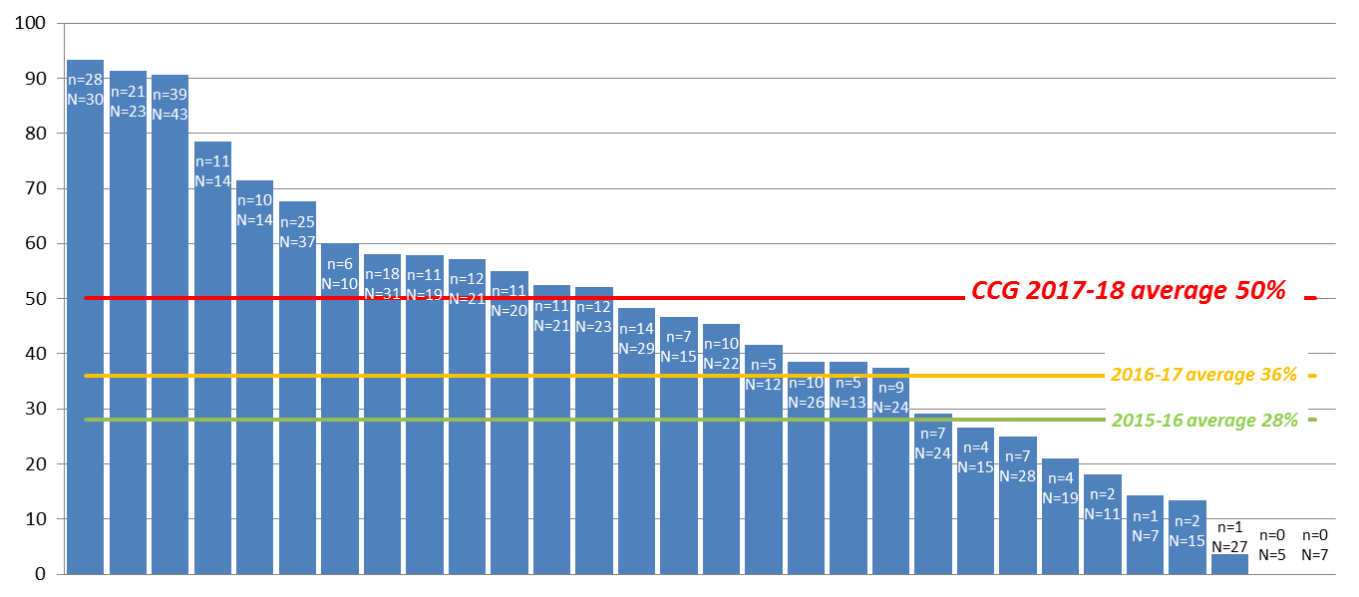
Percentage of episodes with acute kidney injury on discharge summary and Read coded by primary care practice (April 2017 to March 2018).

The audit data demonstrated significant improvements (p<0.05) in all four criteria measured: (1) recording of AKI; (2) medication review; (3) kidney function check; and (4) written information about AKI given to patients. Diagnostic Read coding of AKI increased by 22% from 28% in 2015/2016, to 36% in 2016/2017, and then to 50% in 2017/2018. However, coding was variable between practices, with coding of AKI episodes ranging between 0% and 93% (figure 1).

**Figure 2:**
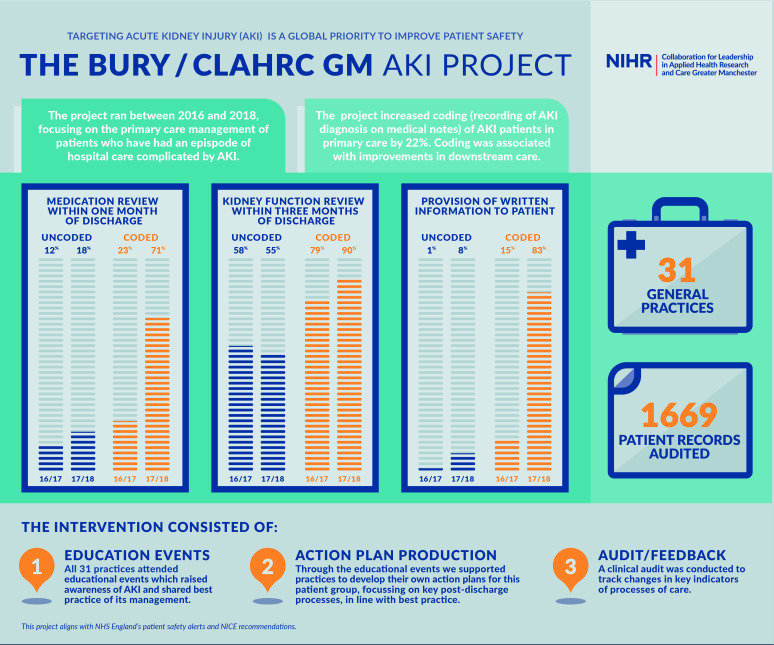
Summary of the intervention showing improvement in Read coding, medication review, kidney function testing and provision of written information.

Coding of AKI on primary care systems was associated with significant improvements in patient management in terms of increases in timely medication review, kidney function monitoring and written information being given to patients. Due to the proportion of episodes of AKI *not* Read coded, we report on the processes of care for both patients who were Read coded with an AKI diagnosis and those who were not (figure 2).

**Figure 3:**
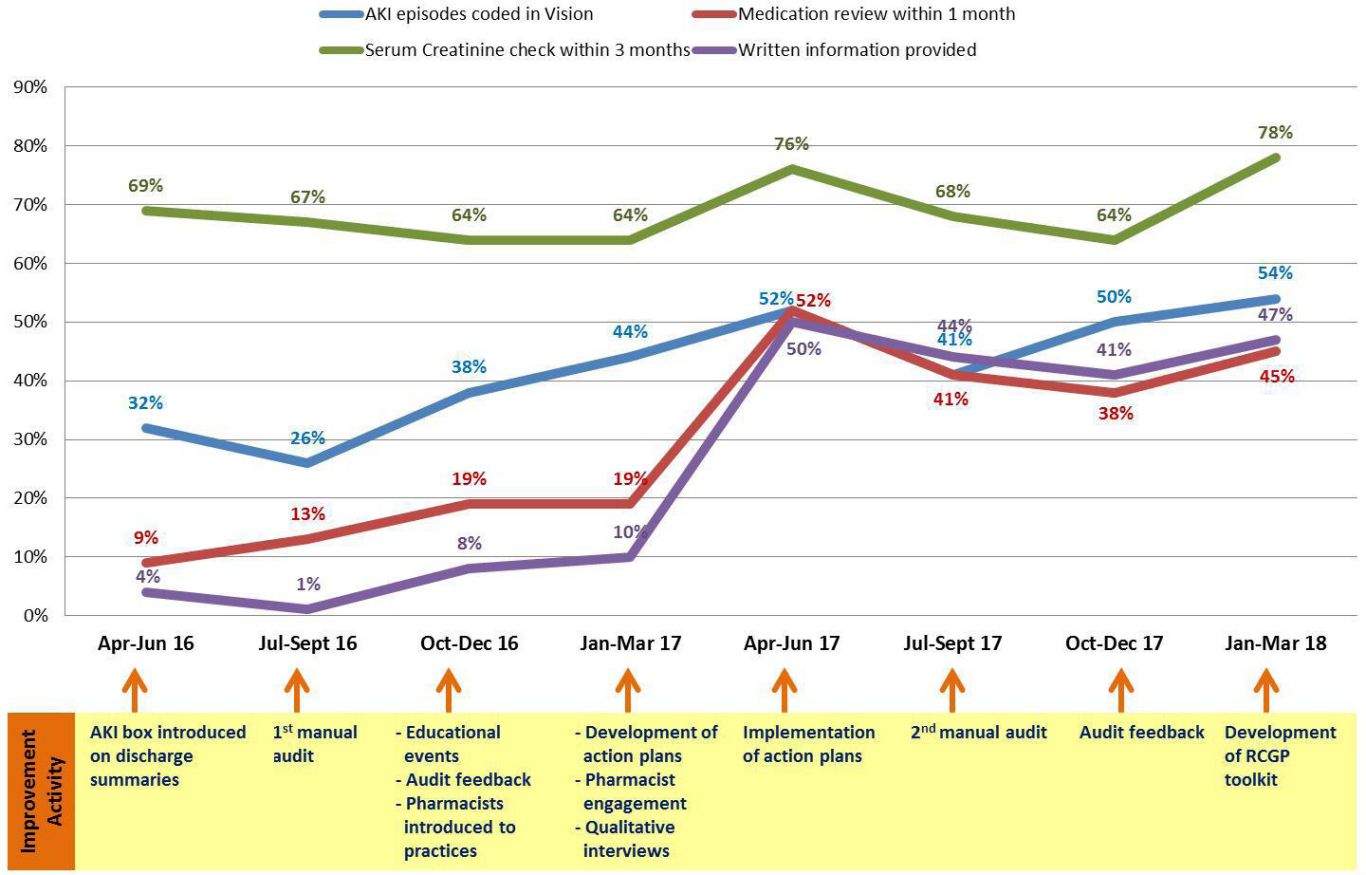
Percentage of episodes with acute kidney injury (AKI) on discharge summary who achieved the key indicators per quarter over the course of the intervention (April 2016 to March 2018).

Figure 3 shows the trends in the four criteria measured, documented alongside the timing of delivery of the various elements of the complex intervention. Further details are provided in appendix 1 of the NIHR CLAHRC GM report.^43^ Improvements in activity followed the first audit/feedback time point, educational events and development of the practice-level action plans, indicating the change in activity was associated with the quality improvement work. The data also suggest improvements were sustained for at least a year following the intervention, although downstream analysis would be required to assess longer term sustainability. It is highly unlikely that provision of written AKI information in particular would have been offered to such an extent without this improvement work, further supporting the impact of this intervention.

#### Medication reviews

For episodes of AKI that were Read coded, evidence of a medication review having been conducted within 1 month of discharge increased from 23% in 2016/2017 to 71% in 2017/2018. In comparison, for episodes that were not Read coded, the rate increased from 12% (2016/2017) to 18% (2017/2018).

#### Monitoring kidney function

For episodes of AKI that were Read coded, evidence of a serum creatinine test having been checked within 3 months of discharge increased from 79% (2016/2017) to 90% (2017/2018), whereas episodes that were not Read coded decreased from 58% (2016/2017) to 55% (2017/2018).

#### Communication with patients

Evidence of written information being given to patients about AKI increased from 15% (2016/2017) to 83% (2017/2018) in those with a Read-coded AKI diagnosis, compared with an increase from 1% (2016/2017) to 8% (2017/2018) in episodes that were not Read coded.

The reduction in frequency of delivering the audit feedback to practices (due to the shift from using an informatics tool quarterly in the original design to annual audit visits with manually collated data) may be expected to impact on the findings, yet the intervention still resulted in significant improvements in downstream clinical care.

### Outcomes

The rates of readmission in NHS Bury CCG in 2017/2018 were 19.4% at 30 days and 29% at 90 days. Despite the significant improvements in primary care processes observed, there was no statistically significant effect on hospital and mortality outcomes on average in the 2 years following the start of the intervention compared with other CCGs served by the same hospital. Furthermore, there was no difference in outcomes between Bury primary care practices that were defined as high performers in terms of Read coding and medication reviews (above average levels in 2017–2018) compared with control group practices. For further details, please refer to the outcome evaluation published in the NIHR CLAHRC GM report.^43^

## Lessons and limitations

The project provides a framework to enable implementation of national guidance that recommends the establishment of AKI registers in general practice and for all NHS providers to “develop an action plan to ensure any relevant resources are used to improve local systems and processes for the care of patients with AKI”.^6 12 30^ The findings from the project suggest that incentivising hospital discharge summaries may be a starting point but that quality improvement activities across the interface into general practice are necessary to improve care for this high-risk patient population.^11^ A multi-centre trial across five hospitals showed AKI incidence rate of 7.8 in every 100 hospital admissions.^19^ However, NICE piloting of AKI indicators revealed low levels of diagnostic coding.^29^ In 2016/2017, an average of 3 patients per practice/year (range 0–9) were assigned the relevant Read codes in general practice following discharge. Our quality improvement intervention in NHS Bury CCG demonstrated that it is possible to address this implementation gap.

Specific evaluation data from the training events were not generated. However, the process evaluation interviews explored participants’ views on all components of the intervention, including the events.^43^

Despite improvements in primary care processes, there was no statistically significant effect on unplanned hospitalisation and mortality in the 2 years following the introduction of the intervention in NHS Bury CCG. Although effects may emerge in the longer term, the findings suggest that the QI intervention successfully engaged a primary care workforce in AKI-related activity but that a higher intensity intervention may be required to improve health outcomes. The high rates of hospital readmission found in our single-centre project resonate with large population-based studies conducted in Scotland and Canada.^17 18^ Sawhney *et al*^17^ noted that acute pulmonary oedema was the most common cause for rehospitalisation following AKI, suggesting that early clinical review and medicines reconciliation is needed for people with heart failure. Placing kidney function tests in clinical context is of paramount importance with recent national guidance emphasising that “clinical euovolaemia is vital to improve symptoms and outcome”.^44 45^

Evaluation of post-AKI care processes in NHS Bury CCG was limited to the use of before-and-after study methods, as no audit data on comparator practices were available. Improvements in AKI care processes could therefore reflect trends occurring nationwide rather than effects of the intervention, although this is unlikely given the magnitude of improvements found and the notable increase in provision of written information to patients. Moreover, information governance meant audit data collection was restricted to patients who were alive and registered at the time of the annual audit (excluding about 60% of patients). Furthermore, although beyond the scope of this primary care quality improvement project, we recognise that ICD-10 coding of AKI is “likely to significantly underestimate true AKI rates”.^46^ Development and utilisation of a real-time audit tool that captures gaps in both the translation of biochemical AKI (ie, AKI warning stage test results) into clinical coding as well as coding across the interface between hospital and primary care is necessary to understand and minimise the impact of the high mortality rate of this patient population.^46^

The strategic level stakeholders at the CCG believed that incentivising this activity was a driver for change, although pay for performance has shown not to be a ‘magic bullet’ in isolation.^47^ Feedback from practices indicated that CCG support and endorsement was, for them, important.^34^ The combination of these two elements, along with alignment with best practice, was a strength of the study design. The role of the practice-based pharmacists was seen as beneficial in the implementation of action plans. Although evidence of the effect on outcomes of pharmacist involvement in medicine reconciliation post-discharge is limited,^48^ investment in practice-based pharmacy roles continues through the new primary care contract.^49^ Finally, although the project demonstrated improvements in provision of written information about AKI and associated risks, future projects would benefit from gaining an understanding of patient and carer experience of care following AKI. To date, patient and public awareness of AKI and kidney health remains limited and engagement in the co-design of future quality improvement interventions warrants greater consideration.^50–52^

## Conclusion

Aligned with national priorities and policy drivers, this project represents an initial step to improve post-discharge care following an episode of illness complicated by AKI.^1 2 4 6 12 15^ The intervention combined incentives and education, with audit and feedback, leading to primary care engagement and significant improvements in four recommended processes of care. Coding of AKI in primary care systems was positively associated with improvements in downstream management.

Our findings, in conjunction with the wider literature, suggest the development and evaluation of a higher intensity intervention that includes targeting people with heart failure may be required to improve health outcomes. To achieve this, greater collaboration across the interface between hospital and primary care is likely to be a necessary element of future AKI quality improvement interventions. In England, the formal introduction of practice pharmacists and incentivised quality improvement activity across primary care networks requires careful consideration.^49 52 53^ As a clinical syndrome that is relevant to a wide range of patients across all health and care settings, AKI quality improvement work may provide an important lens to support a shift from a single disease framework to the development of integrated care systems for people with complex health and social needs.^54–57^

Although all practices participated in three audits, annual manual data collection was costly, was limited to using ICD-coding and in keeping with governance procedures was restricted to the analysis of care processes for patients who were alive at the point of data collection. Further development of a health informatics tool that captures relevant hospital and primary care biochemical and coding data is necessary to map variation and support sustainable real-time quality improvement across the interfaces of care.^41 46 58^
